# The impact of reference pricing and extension of generic substitution on the daily cost of antipsychotic medication in Finland

**DOI:** 10.1186/s13561-014-0009-3

**Published:** 2014-08-19

**Authors:** Hanna Koskinen, Elina Ahola, Leena K Saastamoinen, Hennamari Mikkola, Jaana E Martikainen

**Affiliations:** Research Department, The Social Insurance Institution, Helsinki, 00101 Finland

**Keywords:** Reference price system, Generic substitution, Antipsychotic drugs, Segmented linear regression analysis

## Abstract

**Objective:**

To assess the impact of reference pricing and extension of generic substitution on the daily cost of antipsychotic drugs in Finland during the first year after its launch. Furthermore, the additional impact of reference pricing on prior implemented generic substitution is assessed.

**Methods:**

A retrospective analysis was performed between 2006 and 2010. A segmented linear regression analysis of interrupted time series was used to estimate changes in the levels and trends in the cost of one day of treatment. Of the study drugs, clozapine belonged to generic substitution already at the start of the study period while olanzapine and quetiapine were included in generic substitution alongside with reference pricing in 2009. Risperidone was included in generic substitution in 2008, before reference pricing.

**Results:**

A substantial decrease in the daily cost of all four antipsychotic substances was seen after one year of the implementation of reference pricing and the extension of generic substitution. The impact ranged from -29.9% to -66.3%, and it was most substantial on the daily cost of olanzapine. Also in the daily cost of risperidone a substantial decrease of -43.3% was observed. However, most of these savings, -32.6%, were generated by generic substitution which had been adopted prior.

**Conclusions:**

Reference pricing and the extension of generic substitution produced substantial savings on antipsychotic medication costs during the first year after its launch, but the intensity of the impact differed between active substances. Furthermore, our results suggest that the additional cost savings from reference pricing after prior implemented generic substitution, are comparatively low.

**Electronic supplementary material:**

The online version of this article (doi:10.1186/s13561-014-0009-3) contains supplementary material, which is available to authorized users.

## Background

Pharmaceutical expenditures have been growing rapidly in most European countries exceeding the growth in overall health spending [1]. This is a source of concern to governments and several strategies for reducing or slowing down public expenditure on pharmaceutical products have been discussed and implemented. Essentially, cost containment measures for pharmaceuticals aim to control prices of medicines, or influence demand by implementing financial or professional measures [2],[3].

Reference pricing in drug reimbursement is a widely used cost containment method. It was first formally adopted in Germany in 1989 followed by many of the European Union countries, as well as New Zealand, Australia and Canada among others [4],[5]. In reference-based pricing, pharmaceuticals are classified into clusters based on generic groups, related drug groups or groups according to similar therapeutic effects. The payer then sets a reference price for each cluster based on e.g. either the lowest or the average price of drugs in that group. The reference price defines the maximum reimbursement for all products in the group. Drugs priced at or below the reference price are subsidized while drugs above the reference price require the patient to pay the excess in part or in total. Rather than to constrain the overall pharmaceutical spending, the goal of reference pricing is to control the third-party expenditure on prescription drugs [5],[6].The reasoning behind reference pricing is to stimulate competition and rational decision-making by physicians and consumers [4]. It is not a direct price control, as manufacturers are free to set a price higher than the reference price.

Studies have shown that the introduction of reference price system generates significant savings during the first years of application. Furthermore, reference price systems have generally been associated with a decrease in the prices of the drugs subject to the policy, and more significant price decreases have been observed in the sub-markets where the drugs were already facing generic competition prior to reference pricing [7]. A German study found that the introduction of reference price system reduces prices of the affected products by approximately 7% [8]. In Sweden, drugs covered by the reference price system faced an average price decrease of 19% in the first year after the introduction of the system [9]. Similar results, an estimated price decrease of 18%, were seen in Norway for brand name products while the price reduction for generics was estimated to be about 8%. [10] On the other hand, though the initial average price reduction after the introduction of reference price system in The Netherlands was about 5%, the system also had some negative implications as the prices of several generic drugs were raised towards the maximum reimbursement level [11].

In Finland, generic substitution was introduced in April 2003 and reference pricing in April 2009. Generic substitution systems can vary between countries but basically in generic substitution pharmacies have the right or obligation to substitute the cheapest or close to the cheapest equivalent medicine for a prescribed medicine [12]. As reference pricing and generic substitution were implemented at different times in Finland, we are able to study the additional impact of reference pricing to a market already faced with generic competition. So far, to our knowledge, there have been no studies examining the additional impact of reference pricing to prior implemented generic substitution.

Our specific interest is to study the impact of reference pricing to the daily cost of antipsychotic drugs. Antipsychotics, which are primarily indicated for the treatment of psychotic disorders such as schizophrenia and schizoaffective disorder [13], have in terms of costs been among the fastest-growing therapeutic classes in Finland in the past decades, and the growth was mostly explained by the average cost per one day of treatment [14]. Antipsychotics were not originally included in generic substitution in Finland because of concerns with adherence [15]. However, this decision was later changed and from 2006 onwards antipsychotics were considered to be substitutable, provided they otherwise meet the criteria for substitutability. Clozapine, olanzapine, quetiapine and risperidone are among the most used atypical antipsychotics in Finland [16], and were therefore selected for this study. The selected study drugs were included in generic substitution and reference pricing at different times during the study period. The aim of this study is to assess the impact of reference pricing on the daily cost of antipsychotic drugs in Finland during the first year after its implementation. Furthermore, the additional impact of reference pricing to prior implemented generic substitution is assessed.

## Methods

### Study setting

All permanent residents in Finland are covered by the National Health Insurance scheme, which among other things provides reimbursements for the cost of prescription drugs used in ambulatory care. The rate of the reimbursement varies between drug groups and diagnoses, and during the study years it was either 42% (basic refund category) or 100% plus a fixed co-payment of €3.00 per purchase (higher special refund category) for all of the studied antipsychotics. The higher special reimbursement is available for antipsychotics when used in the treatment of severe psychotic and other severe mental disorders. Regardless of the refund category, the patient’s annual share of the costs cannot exceed a set limit, which was €672.70 in 2010 [17].

Reference pricing was adopted in Finland in April 2009 in tandem with extending the range of medicinal products available for generic substitution. The extension to generic substitution was done by including medicinal products protected by analogy process patent to the scope of substitutable products. The aim of reference pricing was to further enhance the savings generated by generic substitution, which had been adopted in April 2003.

In Finland, it was not possible to grant product patents for medicinal substances prior to 1995, only so called analogy process patents were possible. In order to protect the intellectual property of process patent holders in Finland, the Finnish Medicines Act was amended in 2006 so that pharmaceuticals were excluded from the generic substitution system if they were protected by analogous process patent in Finland and they enjoyed product patent protection in at least five other European Economic Area countries. However, when a generic reference price system was approved by the Finnish government, it was also decided that pharmaceuticals protected by analogous process patent would be included in the sphere of generic substitution (Amendment 803/2008 on Medicines Act [395/1987]). Clozapine was included in generic substitution in January 2006 and risperidone in January 2008. Olanzapine and quetiapine, both protected by analogy process patent, were included in generic substitution alongside with reference pricing in April 2009 (Table 1).

**Table 1:** **Information about the active substances included in the study**

Active ingredient	Marketing authorization	Generic substitution	Reference pricing	Total costs (EUR)		Purchasing individuals (n)	
				2006	2010	2006	2010
Clozapine	1990	1st Jan 2006	1st Apr 2009	4,457,941	5,356,410	7,773	9,227
Olanzapine	1996	1st Apr 2009	1st Apr 2009	37,287,194	21,819,898	20,151	22,857
Quetiapine	2001	1st Apr 2009	1st Apr 2009	23,076,154	20,235,274	29,793	65,351
Risperidone	1994	1st Jan 2008	1st Apr 2009	21,411,458	16,780,347	28,997	37,220

In generic substitution system in Finland, the dispensing pharmacy is obligated to substitute the prescribed medicine to the cheapest, or close to cheapest, product containing the same active substance. Mutually substitutable medicinal products are grouped according to the following criteria: they must have the same active ingredient, the same strength and same pharmaceutical form, and they must be sold in comparable package sizes. The reference price groups are based on the list for substitutable medicinal products compiled by the Finnish Medicines Agency Fimea. A reference price for a group is then set at €1.50 higher than the price of the cheapest product in the group if the cheapest product is priced below €40.00. If the cheapest product is priced at €40.00 or more, the reference price is set €2.00 higher than the cheapest one. Reference prices are subject to changes on a quarterly basis. Patients who wish not to switch to a cheaper medicine are reimbursed according to the reference price, and they must pay the excess themselves. The excess does not count towards the annual limit of out-of-pocket medicine expenses. However, the prescribing doctor may prohibit substitution on medical or therapeutic grounds, in which case the reimbursement is calculated according to the purchase price [17]. Before the reference price system was introduced, both the prescribing physician and the purchasing individual could reject the substitution without affecting the reimbursement rate of the product [18]. Besides the implementations of generic substitution and reference pricing, the reimbursement system in Finland remained substantially unchanged through the study period.

### Data collection

The Social Insurance Institution of Finland maintains a national register, which contains information on reimbursed purchases of medicines. Between 2006 and 2010, the register covered 94-99% of the ambulatory consumption of antipsychotics measured as Defined Daily Doses (DDDs). The data extracted for this study includes information on the patient identity number, the date of dispensing, the total cost of the purchase, Anatomical Therapeutic Chemical (ATC) classification code [19] of the product, and the number of DDDs purchased. The concept of DDD was developed for drug consumption statistics, and it represents the typical daily dose for a drug when it is used for its main indication in adults [19]. The Social Insurance Institution’s register does not include information on the actual prescribed dosage of the drug. Therefore, DDD was used as a proxy for a daily dosage. The DDDs used in this analysis are for the year 2010. The costs used are retail prices inclusive of value-added tax, and they include both the National Health Insurance scheme’s reimbursement part of the price and the patient’s own contributions.

The study data consisted of reimbursed purchases of clozapine (ATC classification code N05AH02), olanzapine (N05AH03), quetiapine (N05AH04) and risperidone (N05AX08) from January 1, 2006 to March 31, 2010.

### Statistical analysis

An interrupted time series design and segmented linear regression analysis were used to estimate changes in the levels and trends in the cost of one day of treatment (daily cost) measured as the cost per DDDs after the introduction of generic substitution and reference pricing. Segmented linear regression analysis of interrupted time series allows us to evaluate longitudinal effects of interventions. It takes account of the pre-intervention level and trend of the daily cost and assesses how much the intervention changes the cost immediately and over time in absolute and relative terms.

Our study material allowed the analysis of the daily cost of clozapine, olanzapine and quetiapine products 39 months before and 12 months after the introduction of reference pricing. Clozapine belonged to generic substitution already at the start of the study period while olanzapine and quetiapine were included in generic substitution at the start of reference pricing. Risperidone was included in generic substitution before reference pricing, and the study material covered 24 months before generic substitution, 15 months after generic substitution but before reference pricing, and 12 months after reference pricing. A mean monthly daily cost was calculated for each of the antipsychotics. Altogether, each dataset had 51 monthly values of the mean daily cost.

The following segmented linear regression model with autoregressive errors was used for clozapine, olanzapine and quetiapine [20],[21]:

1 $$\begin{array}{l} Y_{t}=\beta_{0}+\beta_{1}\times \;\mathrm{tim}e_{t}+\beta_{2}\times \;\mathrm{interventio}n_{t}+\beta_{3}\times \;\mathrm{time}\;\mathrm{after}\;\mathrm{interventio}n_{t}+\nu_{t}\\ \nu_{t}=–\varphi_{1}\nu_{t‐1}–\ldots .–\varphi_{n}\nu_{t‐n}+\varepsilon_{t}\\ \varepsilon_{t}\sim \mathrm{IN}\;\left(0,\sigma^{2}\right) \end{array}$$

In this model *Y*_*t*_ is the mean daily cost of the study medication in month *t*; *time* is a continuous variable indicating time in month at time *t* from the start of the observation period; *intervention* is an indicator for time *t*, coded 0 before reference pricing and 1 after it; *time after intervention* received a value of 0 before reference pricing and was a continuous variable indicating time in months after the change; *β*_*0*_ estimates the baseline level of daily cost per month; *β*_*1*_ estimates the monthly change in daily cost before reference pricing; *β*_*2*_ estimates the level of change in daily cost immediately after the introduction of reference pricing; *β*_*3*_ estimates the change in the trend of daily cost after reference pricing, compared with the monthly trend before reference pricing; and *ν*_*t*_ is an error term consisting of an autoregressive error part *– φ*_*1*_*ν*_*t-1*_*– …. – φ*_*n*_*ν*_*t-n*_ and a random error part *ε*_*t*_. All parameters *β*_*0*_, *β*_*1*_, *β*_*2*_ and *β*_*3*_ that were significant at significance level 0.10 were included in the final models.

As error terms may be correlated in time-series data, the Durbin-Watson test was applied. Autocorrelation was detected in all of the datasets, and thus autoregressive error models were used to estimate the regression parameters with control of autocorrelation. The estimations were done using maximum likelihood methods, which is considered one of the most appropriate approaches for small samples with autocorrelated errors [21],[22]. All significant autoregressive parameters up to 12 months were included in the models, and stepwise elimination with significance level 0.10 was used. In the results section, the autoregressive parameters are assumed fixed, and they are valued according to their estimated values.

The normality and homoscedasticity of the residuals were checked by statistical tests, and graphic analysis of residuals was done to provide information about the consistency. Although statistical tests supported the assumption of homoscedasticity in the olanzapine data, graphical examinations indicated the possibility of the presence of heteroscedasticity: the scatter plot of times and residuals suggested that the variable time might be the source of heteroscedasticity. Therefore, a model without a time variable was also fitted in the olanzapine data.

The following segmented linear regression model with autoregressive errors with two change points was used for risperidone [20],[21]:

2 $$\begin{array}{l} Y_{t}=\beta_{0}+\beta_{1}\times \;\mathrm{tim}e_{t}+\beta_{2}\times \;\mathrm{pr}e_{t}+\beta_{3}\times \;\mathrm{intervention}1_{t}+\beta_{4}\times \;\mathrm{time}\;\mathrm{after}\;\mathrm{intervention}1_{t}+\beta_{5}\times \;\mathrm{intervention}2_{t}+\beta_{6}\times \;\mathrm{time}\;\mathrm{after}\;\\ \mathrm{intervention}2_{t}+\nu_{t}\\ \nu_{t}=–\varphi_{1}\nu_{t‐1}–\ldots .–\varphi_{n}\nu_{t‐n}+\varepsilon_{t}\\ \varepsilon_{t}\sim \mathrm{IN}\;\left(0,\sigma^{2}\right) \end{array}$$

In this model, *β*_*2*_ estimates the pre-effect to generic substitution; *pre*_*t*_ is a dummy variable receiving a value of 1 one month before generic substitution was implemented and a value of 0 at all other time points; *intervention1* refers to generic substitution; and *intervention2* refers to reference pricing.

The estimations, as well as the checking of normality and homoscedasticity of residuals, were done according to the methods described in the case of clozapine, olanzapine and quetiapine.

First, a model without the pre-effect to generic substitution was tried. In that case, a departure from the assumption of homoscedasticity of the residuals was detected. Further investigation identified an outlier as the source of the heteroscedasticity. The outlier was identified as December 2007, indicating that the manufacturers were anticipating the forthcoming generic substitution. Therefore, a dummy variable estimating the pre-effect to generic substitution was constructed for the risperidone model.

The confidence intervals for absolute and relative changes in the daily cost were calculated using bootstrap methods. A bootstrap algorithm based on the theoretical framework of Zhang et al. was constructed [21]. The algorithm consists of five phases. The estimated values of the parameters used below were received from the model fitted to the original data.

1. Fitted values${\hat{Y}}_{t}$ were computed for all t = 1,…, 51:
  3 $$\begin{array}{l} {\hat{Y}}_{t}={\hat{\beta }}_{0}+{\hat{\beta }}_{1}\times \;{\mathrm{time}}_{t}+{\hat{\beta }}_{2}\times \;{\mathrm{intervention}}_{t}+{\hat{\beta }}_{3}\times \;\mathrm{time}\;\mathrm{after}\;{\mathrm{intervention}}_{t},\mathrm{for}\;\mathrm{clozapine},\mathrm{olanzapine}\;\mathrm{and}\;\mathrm{quetiapine}\;\mathrm{and}\\ \mathrm{quetiapine}\;\mathrm{and}\\ {\hat{Y}}_{t}={\hat{\beta }}_{0}+{\hat{\beta }}_{1}\times \;{\mathrm{time}}_{t}+{\hat{\beta }}_{2}\times \;{\mathrm{pre}}_{t}+{\hat{\beta }}_{3}\times \;\mathrm{intervention}1_{t}+{\hat{\beta }}_{4}\times \;\mathrm{time}\;\mathrm{after}\;\mathrm{intervention}1_{t}+{\hat{\beta }}_{5}\times \;\mathrm{intervention}2_{t}\\ +{\hat{\beta }}_{6}\times \;\mathrm{time}\;\mathrm{after}\;\mathrm{intervention}2_{t},\mathrm{for}\;\mathrm{risperidone} \end{array}$$

The estimated values${\hat{\beta }}_{0},{\hat{\beta }}_{1}\ldots ,{\hat{\beta }}_{6}$of the parameters β_0_, β_1_,…,β_6_ were used and only the significant parameters were involved.

- Next three phases were repeated 10 000 times.
- Simulated values${\tilde{\nu }}_{t},$t = 1, …, 51 were drawn from the model
  4 $$\begin{array}{l} \nu_{t}=‐{\hat{\varphi }}_{1}\nu_{t‐1}‐\ldots ‐{\hat{\varphi }}_{n}\nu_{t‐n}+\varepsilon_{t}\;\\ \varepsilon_{t}\sim \mathrm{IN}\;\left(0,{\hat{\sigma }}^{2}\right) \end{array}$$

The estimated values${\hat{\varphi }}_{1},\ldots ,{\hat{\varphi }}_{n}$of the parameters φ_1_, …, φ_n_ were used and only the significant parameters were involved. The root mean square error (RMSE) was used as the estimated value$\hat{\sigma }$of the parameter σ. An exact simulation algorithm as described in Woodfield [23] was used (SAS/IML function ARMASIM).

b. New simulated data${\tilde{Y}}_{t}={\hat{Y}}_{t}+{\tilde{\nu }}_{t}$s , t = 1, …, 51, was created.
c. The model with the same structure as in the model fitted to the original data was fitted to the new simulated data and the new estimated values of the parameters received were stored.
3. The stored collection of the new estimated values is the empirical distribution of the estimated values and was used when confidence intervals were calculated.

All analyses were conducted using SAS 9.2 [24].

## Results

Between January 1, 2006 and March 31, 2010, there were 402,869 purchases of clozapine, 380,680 purchases of olanzapine, 697,026 purchases of quetiapine, and 498,339 purchases of risperidone.

**Figure 1:**
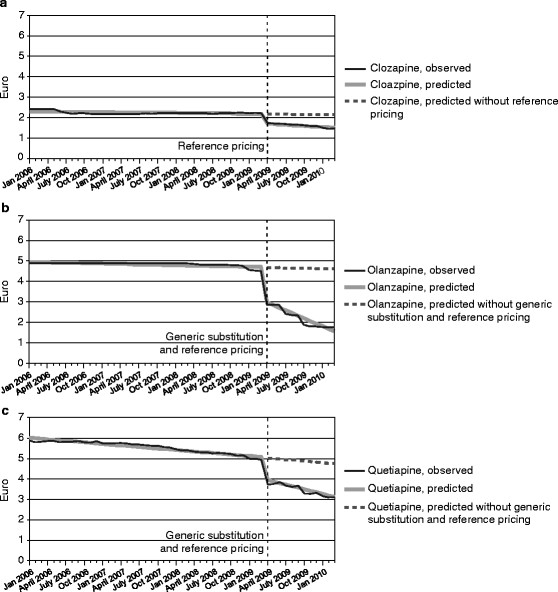
**Daily cost trends of clozapine, olanzapine and quetiapine.** **a** Observed and predicted daily cost of clozapine and the forecast cost had reference pricing not been implemented (EUR). **b** Observed and predicted daily cost of olanzapine and the forecast cost had generic substitution and reference pricing not been implemented (EUR). **c** Observed and predicted daily cost of quetiapine and the forecast cost had generic substitution and reference pricing not been implemented (EUR).

The results of the regression model for clozapine are presented in Table 2. Though the 95 percent confidence intervals include zero, during the generic substitution period the daily cost of clozapine had a downward month-to-month trend and this trend accelerated after reference pricing when significance level 0.10 is used. Immediately after reference pricing was implemented, the level of the daily cost dropped substantially (Figure 1a).

**Table 2:** **Impact of reference pricing on the daily cost of clozapine in Finland**

	Estimate	95% CI	***P***
Level before reference pricing (β_0_)	2.3008	2.2139, 2.3877	<0.0001
Trend before reference pricing (β_1_)	−0.0031	−0.0068, 0.0006	0.0976
Level change after reference pricing (β_2_)	−0.4676	−0.5257, −0.4095	<0.0001
Trend change after reference pricing (β_3_)	−0.0145	−0.0314, 0.0024	0.0905

For olanzapine, a downward month-to-month trend was observed already before generic substitution and reference pricing were introduced. Immediately after the implementation, the level of the daily cost dropped by €1.58, and the downward month-to-month trend accelerated (Table 3, Figure 1b). As the scatter plot of times and residuals suggested the possibility of heteroscedasticity, the consistency of the estimates was checked by omitting time variable from the model. The results proved to be robust.

**Table 3:** **Impact of generic substitution and reference pricing on the daily cost of olanzapine in Finland**

	Estimate	95% CI	***P***
Level before generic substitution and reference pricing (β_0_)	4.9495	4.8113, 5.0877	<0.0001
Trend before generic substitution and reference pricing (β_1_)	−0.0068	−0.0127, −0.0009	0.0257
Level change after generic substitution and reference pricing (β_2_)	−1.5789	−1.6986, −1.4592	<0.0001
Trend change after generic substitution and reference pricing (β_3_)	−0.1229	−0.1470, −0.0988	<0.0001

The results from the regression model for quetiapine are presented in Table 4. The regression curve (Figure 1c) has the same shape as in the case of olanzapine: a downward month-to-month trend before generic substitution and reference pricing were introduced, a substantial drop in the level of the daily cost immediately upon implementation, and an acceleration in the trend after the implementation.

**Table 4:** **Impact of generic substitution and reference pricing on the daily cost of quetiapine in Finland**

	Estimate	95% CI	***P***
Level before generic substitution and reference pricing (β_0_)	6.0130	5.8591, 6.1669	<0.0001
Trend before generic substitution and reference pricing (β_1_)	−0.0248	−0.0311, −0.0185	<0.0001
Level change after generic substitution and reference pricing (β_2_)	−1.0493	−1.1634, −0.9352	<0.0001
Trend change after generic substitution and reference pricing (β_3_)	−0.0506	−0.0757, −0.0254	0.0002

**Figure 2:**
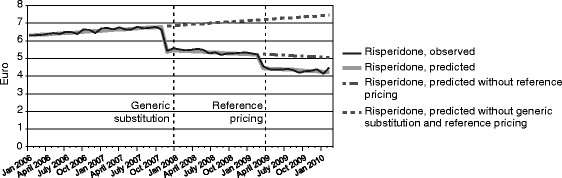
**Observed and predicted daily cost of risperidone and the forecast cost had generic substitution and reference pricing not been implemented (EUR).**

The daily cost of risperidone had a slight but statistically significant upward trend before generic substitution was implemented, but this trend turned downward after generic substitution. Immediately after also reference pricing was implemented, the level of the daily cost of risperidone dropped substantially (Table 5, Figure 2). There was no statistically significant (P = 0.1426) change in the month-to-month trend after reference pricing was implemented, and therefore this factor was eliminated from the model.

**Table 5:** Impact of generic substitution and reference pricing on the daily cost of risperidone in Finland

	Estimate	95% CI	***P***
Level before generic substitution (β_0_)	6.2567	6.1842, 6.3292	<0.0001
Trend before generic substitution (β_1_)	0.0226	0.0178, 0.0274	<0.0001
Pre-effect to generic substitution (β_2_)	−1.4291	−1.5947, −1.2635	<0.0001
Level change after generic substitution (β_3_)	−1.3164	−1.4068, −1.2260	<0.0001
Trend change after generic substitution (β_4_)	−0.0407	−0.0497, −0.0317	<0.0001
Level change after reference pricing (β_5_)	−0.7895	−0.9001, −0.6789	<0.0001

The absolute and relative effects of reference pricing to the average daily cost of the study drugs after one year of application are presented in Table 6. For clozapine, without the implementation of reference pricing the estimated daily cost would have been €2.14. With reference pricing, the estimated daily cost was €1.50, representing an absolute reduction of €0.64 and a relative reduction of 29.9%. For olanzapine, the absolute and relative reductions were even more substantial, €3.05 and 66.3%, respectively. The corresponding numbers for quetiapine were €1.66 and 34.9%. In the case of risperidone, the relative reduction the daily cost after one year of the implementation of reference pricing was 43.3%. However, a substantial part of the savings was generated by generic substitution, and the estimated daily cost of risperidone at the end of the study period would have been €4.99 if reference pricing had not been implemented. That corresponds to a relative change of −32.6% (95% CI −35.2%, −30.0%).

**Table 6:** **Absolute and relative effects of the interventions to the average daily cost, estimated from the regression models**

	Daily cost with intervention/-s	Daily cost without intervention/-s	Absolute change		Relative change	
	Euros	Euros	Euros	95% CI	%	95% CI
Clozapine^a^	1.5014	2.1430	−0.6416	−0.8355, −0.4461	−29.9	−37.6, −21.8
Olanzapine^b^	1.5509	4.6046	−3.0537	−3.3425, −2.7660	−66.3	−70.7, −61.8
Quetiapine^b^	3.0917	4.7482	−1.6565	−1.9443, −1.3670	−34.9	−39.8, −29.7
Risperidone^c^	4.2045	7.4093	−3.2048	−3.4034, −3.0069	−43.3	−44.9, −41.5

^a^Intervention: reference pricing.

^b^Interventions: generic substitution and reference pricing implemented simultaneously.

^c^Interventions: generic substitution and reference pricing implemented separately.

## Discussion

In our study, a substantial decrease in the daily cost of all four antipsychotics was seen after one year of the implementation of reference pricing. The intensity of the impact, however, differed between different active substances. Furthermore, as generic substitution and reference pricing were implemented at different times in Finland, we were able to study the additional impact of reference pricing on prior implemented generic substitution and our results suggest that the additional impact of reference pricing remains comparably small. The result might, nevertheless, be different in other drug classes.

The differences between different active substances can partly be explained by different reimbursement levels for the substances. For example, in 2009, over 80% of olanzapine purchases were reimbursed according to the higher special refund category compared to about 30% of quetiapine purchases [16]. If a patient decides to purchase a product which belongs to a reference price group and is priced above the reference price, his share of the cost in the higher special refund category can be considerably higher than the fixed co-payment of €3.00 per purchase. This might influence patients’ willingness to switch to a reference-priced product, and therefore influence the manufacturers’ willingness to price their products at maximum to the reference price. As the higher special refund category can be interpreted as a proxy for illness severity, it is also possible that the patients purchasing olanzapine have a more serious condition and have therefore more restricted financial resources. This might also influence the manufacturers’ willingness to price their products at maximum to reference price.

When comparing the substances where the implementation of reference pricing had the largest and the smallest impact on costs, olanzapine and clozapine, the differences can partly be explained by the simultaneous entering to reference pricing and generic substitution in the case of olanzapine. The sales volumes can also partly explain the differences. As the consumption of olanzapine was higher than that of clozapine, the incentive for the manufactures of olanzapine to take part in price competition was most probably greater, even though there were only a few manufacturers operating in the market. The low number of generic olanzapine manufacturers can, in turn, be explained by international markets, as in many countries olanzapine was still under patent protection during our study period.

In the case of risperidone, manufacturers anticipated the forthcoming generic substitution and lowered their prices one month before the system was implemented. One reason for this action could be that the manufacturers already operating in the market were trying to control the incentive for new competitors to enter the market. Also, as risperidone had lost its international patent exclusivity already in 2007, it is possible that manufacturers based their pricing decisions in Finland on international price levels. Nevertheless, in our study the additional impact of reference pricing to prior implemented generic substitution remained surprisingly low indicating that generic substitution is effective in promoting price competition. Our results are in contrast to earlier studies which have indicated that reference pricing produces more significant price decreases in the sub-markets where drugs were already facing generic competition prior to reference pricing [7].

For all of the antipsychotics studied, the number of patients purchasing the products increased during our study period. The increase was especially high for quetiapine, for which the number of purchasing individuals more than doubled between 2006 and 2010. This can partially be due to decrease in prices, but also the use of quetiapine for a spectrum of new indications, for example for sleep disorders, can explain the phenomenon. However, as the underlying purpose of reference pricing is to contain pharmaceutical expenditures, potential increases in demand of pharmaceutical products due to lower prices should be studied. Besides this, there are other important issues related to reference pricing which are beyond the scope of this study. For instance, reference pricing has been criticized for potentially adverse effects on the health of those who switch from one product to another, as well as negatively affecting the intensity of pharmaceutical research and introduction of new medicines [25]. However, systematic reviews examining the effects of reference pricing have found no association between reference pricing and health outcomes, though more evidence is still needed [7],[26]. This might be especially important with therapeutic groups such as antipsychotics where patients are often regarded as particularly vulnerable and where medication adherence has been shown to be strongly related to hospitalisations [27],[28].

The decision to include pharmaceuticals protected by analogy process patents to the scope of generic substitution in Finland caused heated debate. For example, the Office of the United States Trade Representative added Finland to its Special 301 report watch list because of concerns about the lack of product patent protection for certain top-selling U.S. pharmaceutical products [29]. It was also claimed that the decision will cause irreversible damage to Finland’s reputation and innovation infrastructure. This would in long term affect negatively the nation’s pharmaceutical expenditures and launching of new and innovative drugs. For example, a study analyzing the effect of price regulation on the launch delay of new drugs in the 1990s found that countries with lower expected prices or small potential sales volumes have fewer launches and longer launch delays [30]. However, so far there is no research evidence that the adopted reference price system would have affected patients’ access to new innovative drugs while cost savings to the society and patients have been demonstrated [31].

Our study is based on a national prescription register. Such a large database gives a comprehensive basis to study the impact of interventions on drug prices. A limitation of this study is that DDDs were used as a proxy of a daily dosage. DDD is a statistical unit of measurement and does not necessarily reflect the prescribed daily doses. Nevertheless, DDD is an internationally well accepted and used unit in drug utilisation studies and therefore the best available measure for this study. Also, an international study found that the DDD system is a reliable tool for standardizing antipsychotic doses in drug utilization research [32]. A further limitation is that the time period in our study only covered the first year after reference pricing was implemented, and, therefore, the effects can only be seen in the short run. However, international experiences have suggested that reference pricing is effective in forcing prices down to the reference price, but there are no incentives for manufacturers to further reduce the prices [33]. Experiences from Germany also suggest that though the prices of drugs included in reference pricing decline, branded drug manufacturers compensate for this by increasing the price of those products that are not subject to the policy [8],[34]. This topic is beyond the scope of this study but warrants future research. In Finland the drug prices are strictly regulated, and increasing the prices of products already on the market is difficult. Increases in prices can, however, be achieved e.g. by launching new products, which in many cases add little innovation but are highly priced [34]. On the other hand, it has been suggested that though reference pricing encourages generic entry and reduces prices, the decline in off-patent drug costs might be even faster in settings where there is no price regulation such as reference pricing [35]. One explanation for this is a so-called ratchet effect: unless justified by increased input costs, low prices will in general be difficult to increase again, and therefore products are priced higher than they would be in an unregulated environment [36]. Intense price competition can also have unintended consequences. It can lead to a downward price spiral which prevents profit making and results in manufacturers withdrawing from the market. In our study, there is no evidence of manufacturers withdrawing from the market because of price spiral either during generic substitution or after reference pricing was implemented. However, the time period of our study only covered the first year of reference price system, and possible long term effects warrant further research [37]. Further research is also needed on whether the decision of the originator brand to take part in price competition or not has an effect on the intensity of the competition. In our data some originator brands took part in the price competition while some did not.

An interrupted time series design is a strong quasi-experimental approach while segmented regression analysis is a powerful statistical method when evaluating longitudinal effects of interventions. One of the greatest strengths of this approach is its comprehensible and graphically intuitive presentation of results. The method is especially well suited for interventions that take place abruptly and it has been widely used in health policy evaluations [20],[38]. However, there are some potential methodological challenges associated with the method. One of the major potential biases for interrupted time series designs are simultaneously occurring interventions. Also seasonal variations in time series are possible. As a rule of thumb, at least 12 time points are needed before and after the intervention in order to detect seasonal variation within the data. As we had 39 data points before reference pricing and 12 data points after reference pricing, we were able to control the seasonal variation. Thus, as no other changes occurred in the reimbursement system or in the health care system during the study period, we can be confident that the observed changes in the daily cost of the four antipsychotic classes were due to the implementation of generic substitution and reference pricing.

In 2009 and 2010, the price competition induced by reference pricing and extended generic substitution generated substantial savings in Finland both in the sales of medicines and the reimbursement expenditures, and the total savings were greatest in the antipsychotics group [31],[39].

## Conclusions

Reference pricing produced substantial reduction of the daily cost of antipsychotic medication in Finland during the first year of application, but the intensity of the impact varied between the active substances. Our results suggest that the additional cost savings from reference pricing that followed the adoption of generic substitution, are comparatively low.

## Authors’ original submitted files for images

Below are the links to the authors’ original submitted files for images. Authors’ original file for figure 1 Authors’ original file for figure 2 Authors’ original file for figure 3 Authors’ original file for figure 4 Authors’ original file for figure 5 Authors’ original file for figure 6 Authors’ original file for figure 7 Authors’ original file for figure 8 Authors’ original file for figure 9 Authors’ original file for figure 10
