# Supplementary figures and images for: The impact of reference pricing and extension of generic substitution on the daily cost of antipsychotic medication in Finland
Source: Health Econ Rev. 2014 Aug 19;4:9. doi: 10.1186/s13561-014-0009-3 (PMC4884034; doi:10.1186/s13561-014-0009-3)

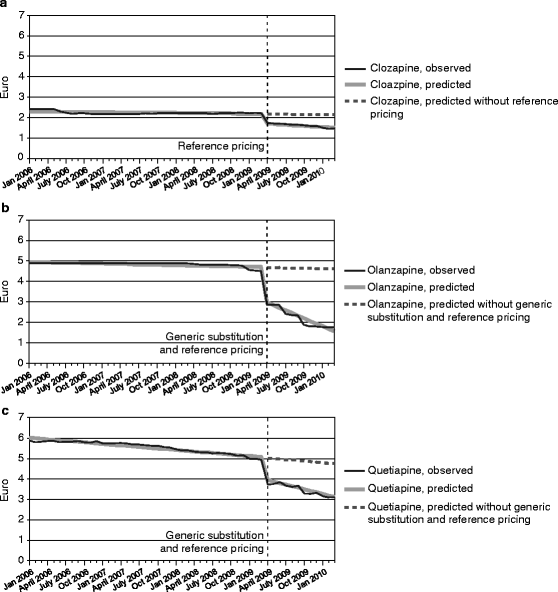

Supplement: Supplementary file 1 — Authors’ original file for figure 1 [file 13561_2014_9_MOESM1_ESM.gif]

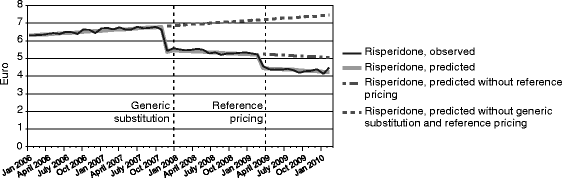

Supplement: Supplementary file 2 — Authors’ original file for figure 2 [file 13561_2014_9_MOESM2_ESM.gif]
