# Supplementary material for: The impact of reference pricing and extension of generic substitution on the daily cost of antipsychotic medication in Finland
Source: Health Econ Rev. 2014 Aug 19;4:9. doi: 10.1186/s13561-014-0009-3 (PMC4884034; doi:10.1186/s13561-014-0009-3)
Supplement: Supplementary file 3 — Authors’ original file for figure 3 [file 13561_2014_9_MOESM3_ESM.docx]

**Table 3** Impact of generic substitution and reference pricing on the daily cost of risperidone in Finland

|  | Estimate | 95% CI | *P* |
| --- | --- | --- | --- |
| Level before generic substitution (β_0_) | 6.2567 | 6.1842, 6.3292 | <0.0001 |
| Trend before generic substitution (β_1_) | 0.0226 | 0.0178, 0.0274 | <0.0001 |
| Pre-effect to generic substitution (β_2_) | -1.4291 | -1.5947, -1.2635 | <0.0001 |
| Level change after generic substitution (β_3_) | -1.3164 | -1.4068, -1.2260 | <0.0001 |
| Trend change after generic substitution (β_4_) | -0.0407 | -0.0497, -0.0317 | <0.0001 |
| Level change after reference pricing (β_5_) | -0.7895 | -0.9001, -0.6789 | <0.0001 |
