# Supplementary material for: The impact of reference pricing and extension of generic substitution on the daily cost of antipsychotic medication in Finland
Source: Health Econ Rev. 2014 Aug 19;4:9. doi: 10.1186/s13561-014-0009-3 (PMC4884034; doi:10.1186/s13561-014-0009-3)
Supplement: Supplementary file 4 — Authors’ original file for figure 4 [file 13561_2014_9_MOESM4_ESM.docx]

Table 1. Information about the active substances included in the study.

| Active  ingredient | Marketing  authorization | Generic substitution | Reference pricing | Total costs (EUR) | | Purchasing individuals (n) | |
| --- | --- | --- | --- | --- | --- | --- | --- |
|  |  |  |  | 2006 | 2010 | 2006 | 2010 |
| Clozapine | 1990 | 1st Jan 2006 | 1st Apr 2009 | 4,457,941 | 5,356,410 | 7,773 | 9,227 |
| Olanzapine | 1996 | 1st Apr 2009 | 1st Apr 2009 | 37,287,194 | 21,819,898 | 20,151 | 22,857 |
| Quetiapine | 2001 | 1st Apr 2009 | 1st Apr 2009 | 23,076,154 | 20,235,274 | 29,793 | 65,351 |
| Risperidone | 1994 | 1st Jan 2008 | 1st Apr 2009 | 21,411,458 | 16,780,347 | 28,997 | 37,220 |
