# Supplementary material for: The impact of reference pricing and extension of generic substitution on the daily cost of antipsychotic medication in Finland
Source: Health Econ Rev. 2014 Aug 19;4:9. doi: 10.1186/s13561-014-0009-3 (PMC4884034; doi:10.1186/s13561-014-0009-3)
Supplement: Supplementary file 5 — Authors’ original file for figure 5 [file 13561_2014_9_MOESM5_ESM.docx]

Table 2a. Impact of reference pricing on the daily cost of clozapine in Finland.

|  | Estimate | 95% CI | *P* |
| --- | --- | --- | --- |
| Level before reference pricing (β_0_) | 2.3008 | 2.2139, 2.3877 | <0.0001 |
| Trend before reference pricing (β_1_) | -0.0031 | -0.0068, 0.0006 | 0.0976 |
| Level change after reference pricing (β_2_) | -0.4676 | -0.5257, -0.4095 | <0.0001 |
| Trend change after reference pricing (β_3_) | -0.0145 | -0.0314, 0.0024 | 0.0905 |

Table 2b. Impact of generic substitution and arefrence pricing on the daily cost of olanzapine in Finland.

|  | Estimate | 95% CI | *P* |
| --- | --- | --- | --- |
| Level before generic substitution and reference pricing (β_0_) | 4.9495 | 4.8113, 5.0877 | <0.0001 |
| Trend before generic substitution and reference pricing (β_1_) | -0.0068 | -0.0127, -0.0009 | 0.0257 |
| Level change after generic substitution and reference pricing (β_2_) | -1.5789 | -1.6986, -1.4592 | <0.0001 |
| Trend change after generic substitution and reference pricing (β_3_) | -0.1229 | -0.1470, -0.0988 | <0.0001 |

Table 2c. Impact of generic substitution and reference pricing on the daily cost of quetiapine in Finland.

|  | Estimate | 95% CI | *P* |
| --- | --- | --- | --- |
| Level before generic substitution and reference pricing (β_0_) | 6.0130 | 5.8591, 6.1669 | <0.0001 |
| Trend before ganeric substitution and reference pricing (β_1_) | -0.0248 | -0.0311, -0.0185 | <0.0001 |
| Level change after generic substitution and reference pricing (β_2_) | -1.0493 | -1.1634, -0.9352 | <0.0001 |
| Trend change after generic substitution and reference pricing (β_3_) | -0.0506 | -0.0757, -0.0254 | 0.0002 |
