# Supplementary material for: The impact of reference pricing and extension of generic substitution on the daily cost of antipsychotic medication in Finland
Source: Health Econ Rev. 2014 Aug 19;4:9. doi: 10.1186/s13561-014-0009-3 (PMC4884034; doi:10.1186/s13561-014-0009-3)
Supplement: Supplementary file 6 — Authors’ original file for figure 6 [file 13561_2014_9_MOESM6_ESM.docx]

Table 4. Absolute and relative effects of the interventions to the average daily cost, estimated from the regression models.

|  | Daily cost  with  intervention/-s | Daily cost  without  intervention/-s | Absolute change | | Relative change | |
| --- | --- | --- | --- | --- | --- | --- |
|  | Euros | Euros | Euros | 95% CI | % | 95% CI |
| Clozapine^a^ | 1.5014 | 2.1430 | -0.6416 | -0.8355, -0.4461 | -29.9 | -37.6, -21.8 |
| Olanzapine^b^ | 1.5509 | 4.6046 | -3.0537 | -3.3425, -2.7660 | -66.3 | -70.7, -61.8 |
| Quetiapine^b^ | 3.0917 | 4.7482 | -1.6565 | -1.9443, -1.3670 | -34.9 | -39.8, -29.7 |
| Risperidone^c^ | 4.2045 | 7.4093 | -3.2048 | -3.4034, -3.0069 | -43.3 | -44.9, -41.5 |

a Intervention: reference pricing.

b Interventions: generic substitution and reference pricing implemented simultaneously.

c Interventions: generic substitution and reference pricing implemented separately.
